# Advances in understanding and treating disorders of consciousness caused by brainstem injury

**DOI:** 10.1186/s41016-025-00411-9

**Published:** 2025-10-20

**Authors:** Qiheng He, Sipeng Zhu, Xiaoke Chai, Tianqing Cao, Nan Wang, Yi Yang

**Affiliations:** 1 Department of Neurosurgery, Beijing Tiantan Hospital, Capital Medical University, Beijing, 100070 China; 2 China National Clinical Research Center for Neurological Diseases, Beijing, 100070 China; 3 Department of Neurosurgery, Aviation General Hospital, Beijing, 100012 China; 4 Chinese Institute for Brain Research, Beijing, 102206 China; 5 Beijing Institute of Brain Disorders, Beijing, 100069 China

**Keywords:** Disorders of consciousness, Brainstem, Arousal, Neurotransmitter, Functional network

## Abstract

Disorders of consciousness (DoC) present significant challenges in clinical neurology, particularly when caused by brainstem injury. The brainstem’s role, especially its ascending reticular activating system (ARAS), is crucial for maintaining arousal, a fundamental component of consciousness. However, the precise mechanisms by which brainstem injuries lead to DoC remain incompletely understood, and treatment options are limited. This gap in understanding hampers the development of effective therapies and impedes clinical management of these conditions. Here, we provide a comprehensive review of the latest research on the anatomical, neurochemical, and network-based mechanisms linking brainstem injury to DoC. We focus on the brainstem nuclei and neurotransmitter systems, such as serotonin from the dorsal raphe nucleus, norepinephrine from the locus coeruleus, and dopamine from the ventral tegmental area, highlighting their roles in arousal regulation and brainstem–cortical communication. Furthermore, we explore how disruptions in connectivity between the ARAS and cortical networks, as revealed by advanced neuroimaging techniques like diffusion tensor imaging and functional MRI, correlate with the severity of consciousness impairment. Additionally, we discuss therapeutic strategies, including pharmacological interventions and neuromodulation techniques, which aim to restore consciousness by targeting these disrupted networks. This review advances the field by synthesizing current knowledge on the brainstem’s role in consciousness and highlighting the potential of targeted therapies to improve patient outcomes. By elucidating the mechanisms underlying DoC caused by brainstem injury, this review provides a foundation for future research to develop more effective treatments, ultimately contributing to better clinical management and recovery strategies for patients with DoC.

## Background

Understanding consciousness and its disorders is essential for managing patients with brain injuries. In contemporary neuroscience, consciousness is characterized by two fundamental dimensions: arousal/wakefulness and awareness, with their intricate interplay still subject to ongoing investigation [1]. According to the recovery process of consciousness disturbance, arousal is the necessary prerequisite for consciousness. The brainstem ascending reticular activating system (ARAS) is recognized for its critical role in arousal, thereby underscoring the significant influence of the brainstem on human consciousness [2].

Disorders of consciousness (DoC) encompass a range of conditions, from coma to unresponsive wakefulness syndrome (UWS) or vegetative state (VS) and to minimally conscious state (MCS) [3]. Coma refers to the absence of both arousal and awareness; only reflexive movements remain. VS/UWS is characterized by the lack of awareness of the environment and the self, accompanied by intermittent wakefulness that may be triggered by tactile, auditory, or painful stimuli. MCS is indicated by fluctuating and reproducible signs of consciousness, such as visual pursuit, localization to noxious stimulation, or command following [4]. These conditions carry profound implications for patient care and quality of life. The etiology of DoC is diverse, encompassing traumatic brain injury (TBI), intracranial hemorrhage, ischemic stroke, and hypoxic-ischemic brain damage (HIBD), each with distinct mechanisms of injury and pathogenesis sites [1].

**Fig. 1:**
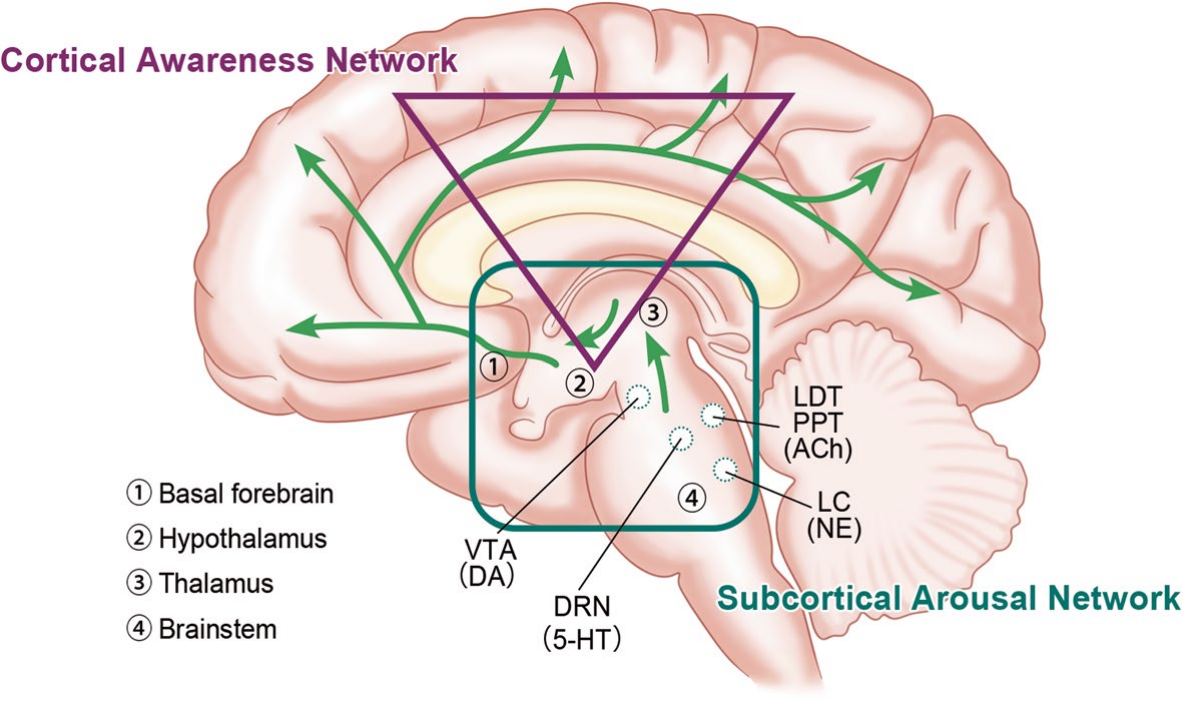
The role of the brainstem on human consciousness. The brainstem, together with the thalamus, hypothalamus, and basal forebrain, constitutes the ascending reticular activating system (ARAS), namely the subcortical arousal network. The activity of this network is closely associated with various arousal nuclei and corresponding neurotransmitters within the brainstem, including norepinephrine (NE) from the locus coeruleus (LC), serotonin (5-HT) from the dorsal raphe nucleus (DRN), dopamine (DA) from the ventral tegmental area (VTA), and acetylcholine (ACh) from the laterodorsal and pedunculopontine tegmental nucleus (LDT/PPT). These neurotransmitters activate subcortical structures via ARAS projection and further act on the distal cortical conscious network. The connectivity within and between the subcortical arousal network and the cortical awareness network jointly supports human consciousness

Notably, brainstem lesions, resulting from infarction, hemorrhage, or diffuse axonal injury (DAI), are particularly likely to induce disturbances in consciousness [2, 5, 6]. The unique characteristics of these patients necessitate a deeper exploration into the mechanisms by which the brainstem sustains consciousness, which is essential for a more profound understanding of the potential pathogenesis and for the development of improved treatment strategies. This review aims to provide a comprehensive overview of the current research status and progress on the relationship between the brainstem and DoC, integrating insights from anatomy, neurobiochemistry, brain networks, and therapeutic discoveries to better elucidate the pivotal role of the brainstem in the maintenance and recovery of consciousness (Fig. 1).

## Microscale perspective of brainstem on consciousness

### Structural and functional significance of the brainstem

The brainstem, a crucial component of the central nervous system, serves as the conduit between the spinal cord and the diencephalon. It exhibits an irregular columnar structure, comprising the medulla oblongata, pons, and midbrain from the bottom up [7]. Beyond its vital role in sustaining life functions, the brainstem is susceptible to injuries that can lead to acute or chronic disruptions in consciousness. The ARAS, a network of neurons and fibers within the brainstem, is pivotal in regulating sleep–wake cycles, attention, and consciousness levels [8]. Key nuclei within the pontine and midbrain, including the locus coeruleus (LC), dorsal raphe nucleus (DRN), pontine nucleus (PO), parabrachial nuclei (PB), pedunculopontine tegmental nucleus (PPT), and laterodorsal tegmental nucleus (LDT), work in concert with the thalamus, hypothalamus, and forebrain to constitute the complete ARAS [7]. This system facilitates the transmission of brainstem activity to the cerebral cortex through neural projections and neurotransmitter release, thereby promoting and maintaining biological arousal.

#### Early animal experiments on arousal

Historically, in 1949, Magoun and his colleagues observed that cats with transection at the pons–midbrain junction exhibited coma symptoms with low-amplitude, synchronous electroencephalograph (EEG) rhythms. Electrical stimulation of the brainstem reticular formation produced arousal signals, while lesions in the midbrain tegmentum abolished this cortical response, underscoring the ARAS as the neural basis for arousal [9, 10]. Subsequent studies, particularly in animal models, have increasingly highlighted the tegmentum’s regulatory influence on arousal, demonstrating that modulation of this area significantly affects wakefulness levels [11].

#### Advanced neuroimaging on pontine tegmentum

Advanced neuroimaging techniques have enabled the study of brain changes in DoC patients, identifying key brainstem nodes that disrupt arousal and consciousness [12]. Parvizi and Damasio [13] investigated 4.7 T magnetic resonance imaging (MRI) from 47 patients with brainstem stroke, identifying that lesions centered in the upper pontine tegmentum were crucial for maintaining consciousness, even in the absence of midbrain damage. Additionally, Fischer et al. [14] employed voxel-based lesion-symptom mapping with functional MRI (fMRI) to compare coma-inducing brainstem lesions with control lesions, pinpointing a 2 mm^3^ region in the pontine tegmentum, ventral to the LC and medial to the PB, as most closely associated with coma. In particular, a hierarchical cluster analysis of blood oxygen level-dependent (BOLD) signals revealed that the pontine tegmentum and caudal midbrain area persistently formed an identical cluster, suggesting their homologous functional attribute in consciousness perturbation [15]. Conversely, the thalamus’s role in the arousal circuit was also scrutinized. In patients with thalamic stroke, severe arousal impairment was associated with lesions involving the midbrain and/or pontine tegmentum, whereas isolated thalamic lesions did not significantly affect arousal, indicating that the most critical structures for human arousal were beyond the thalamus [16].

### Nuclei and neurotransmitters within ARAS

While the anatomical impact of specific brainstem regions on consciousness has been extensively studied, the intricate functions of nuclei within the ARAS at the microscopic and biochemical levels are equally pivotal. These nuclei engage in complex neural circuits through projections and neurotransmitters, affecting the homeostasis of brain function. Anatomically, long-term coma is associated with bilateral damage to the upper pontine tegmentum or both the upper pontine and midbrain tegmentum [17]. Autopsy studies of patients with fatal brain injuries have revealed varying degrees of neuronal damage around these areas, particularly a 17% reduction in serotonergic neurons in the DRN and a 29% decrease in noradrenergic neurons in the LC [18].

#### Serotonergic neurons in the DRN

Serotonin (5-HT), released by DRN neurons, is a critical monoamine neurotransmitter with a contradictory and controversial role in the awakening process. Ito et al. [19] discovered a causal relationship between DRN neuron firing and cortical activity. Specifically, optogenetic stimulation of 5-HT neurons at 20 Hz significantly enhanced wakefulness in mice. It was also demonstrated that CO2-rich solutions applied to DRN 5-HT neurons could elicit arousal, an effect negated by acute pharmacological or optogenetic inhibition of 5-HT neurons [20]. In contrast, evidence from Oikonomou et al. [21] in zebrafish and mice studies harvested the opposite conclusion. These conflicting results leave the role of 5-HT in arousal disturbance still a mystery, potentially due to the diversity and heterogeneity of the DRN 5-HT neuronal subpopulation and parallel pathways controlling distinct physiological responses and brain functions [22, 23].

#### Noradrenergic neurons in the LC

Noradrenergic neurons in the LC, the sole source of norepinephrine in the brain, are vital for regulating mood, attention, and motivation [24]. Cortical EEG and optogenetic studies indicate heightened LC neuronal activity during wakefulness [25]. By utilizing optogenetic tools, Sara [24] demonstrated that long-term light suppression in LC reduced arousal, and the transition to awakening depended on the transmission of norepinephrine, underscoring a robust correlation between LC neuronal activity and wakefulness intensity.

#### Cholinergic neurons in the PPT and LDT

The PPT and LDT were two major cholinergic nuclei in the brainstem. However, stimulation of cholinergic neurons was found to have little effect on awakening; instead, selective photogenetic activation of PPT or LDT could only affect the sleep cycle [26, 27]. Surprisingly, the selective activation of glutaminergic cell populations in PPT produced definite cortical activation and behavioral arousal, which explains the specific regulatory role of chemical transmitters in the arousal process [27].

#### Dopaminergic neurons in the ventral tegmental area

Beyond the pontine tegmentum, additional neurons have been identified as regulators of arousal levels. The significant role of the ventral tegmental area (VTA) dopaminergic (DA) neurons in modulating wakefulness has been the subject of extensive investigation. A fiber photometry study unveiled a dependency relationship between the activity of VTA DA neurons and the arousal state in mice [28]. In genetically engineered mice, selective optogenetic stimulation of VTA DA neurons yielded behavioral and EEG evidence of arousal, indicating a transition from an unconscious state to wakefulness [29]. Moreover, the application of DA receptor antagonists could eliminate the awakening effects induced by the activation of VTA DA neurons [30]. Notably, the DRN also contains DA neurons whose activity peaks during wakefulness and promotes the sleep-to-wake transition [31].

In summary, various nuclei and neurotransmitters within the brainstem are inextricably linked to arousal. The identification of a specific class of neurons that determine the level of consciousness may not be feasible due to the diverse physiological properties of even a single neuron that influence different brain functions [11]. Furthermore, the modulation of neural activity through the release of different neurotransmitters and their interaction is still sophisticated, which remains a complex and unresolved matter requiring further elucidation.

## The relationship between the brainstem and brain networks in consciousness

While ARAS is instrumental in generating and supporting arousal, its activation alone is insufficient in the comprehensive experience of consciousness. Advanced neuroimaging modalities, including fMRI and diffusion tensor imaging (DTI), have been indispensable for examining brain function in various abnormal states. These techniques provide insights into structural and functional connectivity, decode the intricate behaviors of brain activity, and facilitate the exploration of pathogenesis and therapeutic avenues [12]. Based on this, the modern neuroscientific perspective on DoC has shifted from a single, local perspective to global information processing and integration. This transition has given rise to the concept of brain networks, highlighting the interaction and connectivity patterns between diverse brain regions [32]. The communication between the brainstem, subcortical, and cortical regions contributes to the formation of distinct brain networks that support the coherence of cognition, emotion, and motivation and ultimately the human mind and behaviors [33]. Therefore, with the deepening of research, the emergence and progression of consciousness require not only the independent activation of various functional networks in the brain but also effective connections within and between these networks [34].

### Subcortical arousal network

ARAS is a collection of fiber projections connecting the brainstem with the subcortical structures of the hypothalamus, thalamus, and basal forebrain. In other words, the connectivity of these nodes within ARAS is an important component supporting the upward activation of ARAS [35]. Disconnection of these pathways, such as those from the brainstem to the thalamus or basal forebrain, can result in DoC [36]. DTI offers a unique capability to reconstruct and visualize the trajectory and structure of white matter fibers by assessing the dispersion anisotropy of water molecules in tissues [37]. Characterizing the projection, association, and commissural pathways of the subcortical arousal network will allow the understanding and classification of preserved pathways in patients with DoC to identify potential patterns of damage [35].

#### The integrity of white matter

Multiple abnormal white matter fiber deficits have been found in DoC patients with different states of consciousness, and these injuries were significantly correlated with the clinical manifestation of impaired consciousness [38]. More specifically, the fractional anisotropy, tract number, and tract volume values derived from DTI represent the directivity of water diffusion, the number of streamlines, and the voxel number of nerve tracts, respectively. Their decline indicates the injury of white matter integrity [39]. In patients with DoC of various etiologies, such as intracerebral hemorrhage [40], TBI [41], and HIBD [42], the integrity of ARAS was damaged to varying degrees, and these DTI characteristics were correlated with the severity of consciousness impairment. Despite the great heterogeneity of different causes of DoC and injury sites of ARAS, we can still conclude that the integrity of white matter fiber, namely the damage to the axons, affects the normal activity of consciousness.

#### The model of brainstem injury

To delve into the mechanisms by which brainstem lesions lead to coma, researchers injected the vasoconstrictor endothelin-1 into the brainstem to construct a model of brainstem tegmental ischemic injury [39]. T2-weighted MRI and histopathology confirmed that such intervention resulted in focal infarcts within brainstem awakening nuclei but retained the diencephalon, basal forebrain, and cortical structures. In the hyperacute phase of recovery from coma, eigenvector centrality mapping revealed the enhanced longitudinal connections between the awakened nuclei and subcortical structures, especially significant differences of connectivity in the reticular thalamus,

nucleus accumbens, nucleus basalis of Meynert, and striatum. These results highlight the contribution of increased subcortical arousal network connectivity in the recovery from brainstem-induced coma.

#### The combination of injury patterns

It is increasingly recognized that coma is not merely the result of injury to a single arousal nucleus but rather a consequence of combined injuries within the subcortical arousal network. Studies using susceptibility-weighted imaging in comatose patients with severe TBI have demonstrated a relationship between the recovery of consciousness and combination patterns of microbleeds in the awakening nuclei [43]. The midbrain, thalamus, and pons emerged as common sites of microbleeds, with the pontine tegmentum and VTA being particularly vulnerable, which were consistent with the fragile sites of coma described above.

#### The inner connectivity of ARAS

To clarify the connectivity of inner nodes within ARAS in DoC patients, Snider et al. [44] performed high-angular-resolution DTI in patients with acute severe TBI. Compared to matched controls, probabilistic tractography has shown a reduction in ascending arousal axonal pathways connecting the brainstem tegmentum to the hypothalamus, thalamus, and basal forebrain. While the connectivity between brainstem-hypothalamus and brainstem-thalamus was significantly reduced, the brainstem-forebrain connectivity showed no difference. The implications of these results suggest that while the brainstem-forebrain connection may not be essential for the emergence of consciousness, its preservation could be beneficial for the recovery from DoC. This observation points to the possibility that distinct mechanisms underlie the production, maintenance, and recovery of consciousness following injury, with the specific regulatory patterns yet to be fully elucidated. Moreover, the recovery from DoC may involve either the repair of reversible damage or the reorganization of residual pathways post-irreversible damage. The structural connectivity analysis provided by DTI, however, does not discern the reversible or incomplete damage [44], which constrains the exploration of conscious rehabilitation.

### Cortical awareness network

As mentioned before, consciousness is described as a combination of two components: arousal and awareness. Arousal refers to the degree of awakening, while awareness can be divided into awareness of the environment and awareness of the self, predominantly governed by cortical functions [34]. Specifically, the external awareness network mainly consists of the bilateral dorsolateral prefrontal cortex (DLPFC) and posterior parietal cortex (PPC), which are important architectures of the frontal-parietal network (FPN). Its activity is closely related to attention, response, and action, drawing human attention to environmental stimuli [45]. In contrast, the internal awareness network comprises the posterior cingulate cortex (PCC), precuneus (PCu), anterior cingulate cortex (ACC), and medial prefrontal cortex (mPFC), which are the main regions of the default mode network (DMN) [46]. This functional network is mainly active in the resting state and is closely related to self-mental activities [47]. Effective connections within and between these cortical functional networks give birth to the content of awareness, and their connections to the subcortical arousal network are thought to be necessary to support sustained awake activities and conscious processing [35, 48].

#### The connectivity to awareness network

In comatose patients, the damaged cortex may remain static, but consciousness may be restored as long as a sufficient amount of subcortical projections is retained to reactivate the cortex through ascending neural activity from subcortical awakening nuclei [49]. The upper pontine tegmentum, a key brainstem region controlling arousal, gives rise to thalamo-cortical neuronal connectivity [50]. As expected, this region has been found to have extensive connections to cortical networks in healthy individuals, supporting internal and external awareness. In patients with MCS or VS/UWS, functional connectivity between the pontine tegmental and caudal midbrain regions to the cortical awareness network was significantly reduced. Notably, as DoC severity increased, the strength of these functional connections diminished [15]. This indirectly validates the impact of brainstem-cortical connectivity on consciousness and offers a potential neuroimage-based biomarker for the clinical management of patients with DoC.

#### The connectivity to salience network

On the other hand, Fischer et al. [14] demonstrated that the brainstem coma-causing site was functionally connected to the anterior insula (AI) and pregenual anterior cingulate cortex (ACC), covering the distribution of the brain’s von Economo neurons. These neurons, with their extensive dendritic reach across all cortical layers, facilitate rapid information integration and transmission, acting as fast relay stations in the switching process between brain networks [51, 52]. Coincidentally, AI and ACC are the main components of the salience network (SN). Based on chronometric techniques and Granger causality analysis in task-based fMRI, SN was found to play a causal role in the conversion between FPN and DMN [53]. These results have important implications for the transformation of activity between large-scale brain networks involved in consciousness.

### Neurotransmitter systems

The brainstem neurons exert a profound influence on the cerebral cortex and subcortical regions by releasing diverse neurotransmitters. These chemical messengers interact with a heterogeneous distribution of receptors across the cerebral cortex, effectively modulating the transmission and propagation of electrical impulses. By regulating the excitability and discharge rate of cells, neurotransmitters drive synaptic plasticity and alter neural states, thereby facilitating the formation of complex neural network communication and interaction [54]. In particular, the density and distribution patterns of neurotransmitter receptors are consistent with the structural and functional connectivity of the human brain, impacting neural oscillation dynamics and normal brain function [55]. A deeper exploration of the brain’s neurotransmitter systems is crucial for understanding the brain’s role in the production and maintenance of consciousness and for developing new treatment strategies for DoC.

#### Serotonergic and noradrenergic projections

The DRN-derived 5-HT is projected upward to almost all cortical and subcortical regions, mainly including the ventral striatum, hippocampus, amygdala, and cortical structures dominated by ACC, mPFC, and PCC [56], which are key regions of the DMN. This result is also supported by univariate functional connectivity and graph-based analysis, where a functional integration of the serotonergic midbrain nucleus and the DMN was found [33]. Concurrently, the functional correlation of the noradrenergic LC with the executive control network (ECN) was also observed. It is demonstrated that the ECN is part of the large-scale FPN and is involved in the awareness of external information [57]. The LC projects extensively throughout the neocortex and subcortical regions, particularly the frontal and cingulate cortices, where the density of norepinephrine receptors is the highest [33]. In addition, virus tracking techniques confirmed that LC-NA neurons project directly to the ventrolateral preoptic region (VLPO). In the LC-VLPO neural circuit, sleep-active neurons were inhibited by the action of the NA α2 receptor, and awake-active neurons were activated by the NA α1 and *β*-receptors, which allowed them to regulate arousal synergistically and sophisticatedly [25].

#### VTA as a connectivity hub

In vivo 7 T resting-state fMRI data analysis has identified the VTA as a hub node for extensive connectivity between subcortical arousal and cortical awareness networks [35]. It is demonstrated that DA is diffusely released from the VTA to almost all cortical structures, thus mediating macro-level brain network function [54]. In healthy individuals, the VTA demonstrates robust functional connectivity with both subcortical and cortical regions, with the strongest connections observed with cortical DMN nodes [33, 58]. In cases of pharmacological sedation or pathological DoC, the VTA has been found to be disconnected from major DMN nodes, such as the PCu/PCC. Importantly, the strength of VTA-PCu/PCC connectivity correlates significantly with consciousness level, suggesting its potential as an indicator of residual consciousness and a biomarker for clinical prognosis [59].

## Therapeutic approaches

### Pharmacological interventions

Disruptions in the brain’s neurotransmitter systems in DoC suggest a potential role for neural mediators to restore synaptic conduction and reorganize the homeostasis of brain functional networks [60]. Amantadine, a DA agonist, has demonstrated neuroprotective and neuroactivating effects, enhancing neurotransmission in dopamine-dependent circuits crucial for wakefulness [61]. A landmark placebo-controlled trial by Giacino et al. [62] involving 184 patients with chronic DoC following severe TBI reported faster recovery with amantadine regardless of the disease duration and conscious state at enrollment, establishing the potential for neurostimulant substitution in DoC rehabilitation. Owing to its contribution to hastening recovery and reducing disability, amantadine was recommended by the American Academy of Neurology in the practice guideline for DoC in 2018 [3]. Subsequent research has also confirmed amantadine’s therapeutic effects in DoC patients with multiple pathogenic factors [63–65], although this rehabilitative effect might disappear after discontinuation of treatment [62]. In addition, Spindler et al. [59] verified that methylphenidate, a dopaminergic and noradrenergic agonist, could increase the VTA–PCu/PCC connectivity and restore the dopaminergic brainstem connection in a controlled trial involving 12 patients with TBI/DAI.

On the other hand, GABAergic drugs like zolpidem seemed to achieve limited improvement (4.8% responders, 4/84) in DoC patients, with recommendations for its use restricted to non-brainstem injuries due to the lack of cerebral perfusion changes post-medication [66, 67]. Since its effect on consciousness improvement and functional recovery is transient and non-universal, candidate patients should be properly screened and carefully considered in clinical application [4].

### Neuromodulations

#### Noninvasive neuromodulation techniques

Transcranial magnetic stimulation (TMS) and transcranial direct current stimulation are widely utilized non-invasive brain stimulation techniques, demonstrating favorable effects on clinical behavioral performance in DoC patients [4]. While an increase in nerve conduction velocity and cortical activation of the ARAS has been reported following repetitive TMS in VS patients [68], the main targets for these interventions are the DLPFC or primary motor cortex to enhance cortical activation and improve consciousness [69].

Median nerve stimulation (MNS) leverages the communication between the median nerve spinal segment and the ARAS to activate cortical connectivity and promote consciousness recovery [69, 70]. A multicenter randomized controlled trial for acute coma following TBI demonstrated that MNS was more effective than spontaneous recovery in improving consciousness and prognosis [70], which also provided a possible intervention for DoC patients with other etiologies or in prolonged disease.

The vagus nerve can directly regulate the activity of the brainstem through the solitary tract. It was demonstrated that after long-term vagus nerve stimulation (VNS), the basal discharge rate of DRN and LC was significantly increased, resulting in the transmissions of serotonergic and noradrenergic neurotransmitters and activation of cortical and subcortical function [71, 72]. Transcutaneous VNS (taVNS) produces a similar effect by stimulating the auricular branch of the vagus nerve at the ear concha, with the advantages of being non-invasive, economical, and available [73]. Studies have reported the efficacy responses of taVNS on the upgrade of consciousness level [74, 75]. However, it seems that taVNS only makes a difference in MCS patients, with its effectiveness in VS patients being less satisfactory and requiring further validation [76, 77].

#### Invasive neuromodulation techniques

Deep brain stimulation (DBS) is an invasive technique with promising potential to improve DoC by modulating neural pathways through implanted electrodes into deep brain structures [78]. Targets of DBS for DoC include the thalamic central lateral nucleus, center median-parafascicular complex, and other structures integral to the ARAS projection pathways [79, 80]. DBS can enhance consciousness levels by activating the neural circuitry as long as the reticular formation, thalamus, and cortex connectivity are partially intact [81]. Compared with conservative treatment, DBS significantly improved the 1-year rate of consciousness recovery in patients with DoC, particularly in patients with MCS [82]. Although standard protocols and evaluation methods are still in the exploratory stage, more personalized stimulus parameters and closed-loop modulation strategies are needed to achieve future precision medicine treatments [80].

Spinal cord stimulation (SCS), another invasive approach, activates brainstem structures through the upper cervical spinal cord and influences broader brain areas [83]. SCS has shown an estimated effectiveness rate of 30% for VS and 60% for MCS patients [81, 84]. A preliminary report of SCS focused on DoC patients with brainstem hemorrhage suggested potential benefits for neurological rehabilitation and consciousness recovery [85], though further research is warranted given the limited sample size. Monitoring of hemodynamic responses based on functional near-infrared spectroscopy showed that SCS enhanced remote connectivity of the brainstem-thalamic-cortex pathway in a frequency-specific manner to promote consciousness recovery by improving cerebral blood supply and information communication [86].

To sum up, the therapeutic approaches for DoC are diverse, with the main goal of restoring subcortical-cortical neural circuit connections to activate and regulate brain networks for consciousness improvement. However, the common problems of various studies are small sample sizes, nonuniform treatment protocols, and uncertainty of curative effect. Future research should focus on standardized randomized controlled trials to identify optimal treatment methods and parameters, enhancing evidence-based medicine and improving clinical therapeutics for DoC.

## Limitations and prospects

The involvement of the brainstem’s ARAS nuclei in modulating consciousness is multifaceted, with each nucleus possessing distinct neurochemical properties. The neurotransmitters released by these nuclei act on a wide range of cerebral cortex and subcortical regions through different projections, affecting the plasticity of neural synapses and modulating the function of neural networks jointly. While these systems offer valuable insights into the potential pathogenesis and therapeutic targets of DoC, they remain delicate and complex. The modulation of consciousness can vary significantly even among neurons with identical chemical properties due to differences in their projections, and further complexity arises from the interactions among different subsets of neurons [28, 87]. For example, VTA neurons releasing dopamine, GABA, and glutamate can independently influence behavior and function through long-range projections, and there are local connection loops among them that affect each other’s activity levels [87, 88], which add to the complexity of the involvement of brainstem nuclei and brain neurotransmitter systems in consciousness.

Deciphering brain networks by advanced neuroimaging technology with high spatial resolution and electrophysiology technology with high temporal resolution is helpful in delineating the relationship between the brainstem and consciousness. Modern neuroscience has been moving towards decoding brain networks and consciousness in multi-dimensional ways, such as spatiotemporal, dynamic, and causal perspectives. Munn et al. [89] identified a precise time-locked relationship between the low-dimensional, dynamic, and topological features of cerebral cortical and brainstem ARAS activity using high-resolution 7 T resting-state fMRI data. The dynamic reconfiguration of the macroscopic network and energy landscape provides us with a further understanding of the ARAS’s regulatory role in cortical and conscious functions. The integration of neuroimaging and neurophysiology in the field of DoC enhances our comprehension of pathogenesis and neuroplasticity following brain injury. It aids in the exploration of potential neurobiomarkers for clinical decision-making and patient prognosis, with the ultimate goal of providing individualized treatment to improve clinical outcomes in patients with DoC.

At present, studies on DoC caused by brainstem injury are still limited, often constrained by small sample sizes and a dearth of treatment and prognostic insights. The scarcity of research is primarily attributed to the uniqueness of patients with brainstem injury, characterized by etiological heterogeneity, variable disease courses, and diverse lesion sites. The severity of the disease, poor level of consciousness, rare therapeutic interventions, high mortality rates, and difficulties in conducting examinations and treatments collectively restrict the feasibility of in-depth research. In the future, with the improvement of first aid and monitoring facilities, large-scale and multicenter studies on DoC patients with brainstem injury will be conducted to elucidate the disease causes, progression, and outcomes, so as to explain the functional interactions of the brainstem within the global brain network, ultimately contributing to novel therapeutic discovery and improved patient prognosis. 

## Data Availability

Not applicable.

## Abbreviations

DoC: Disorder of consciousness
ARAS: Ascending reticular activating system
UWS: Unresponsive wakefulness syndrome
VS: Vegetative state
MCS: Minimally conscious state
TBI: Traumatic brain injury
HIBD: Hypoxic-ischemic brain damage
DAI: Diffuse axonal injury
LC: Locus coeruleus
DRN: Dorsal raphe nucleus
PO: Pontine nucleus
PB: Parabrachial nuclei
PPT: Pedunculopontine tegmental nucleus
LDT: Laterodorsal tegmental nucleus
EEG: Electroencephalograph
MRI: Magnetic resonance imaging
fMRI: Functional MRI
BOLD: Blood oxygen level-dependent
VTA: Ventral tegmental area
DA: Dopaminergic
DTI: Diffusion tensor imaging
DLPFC: Dorsolateral prefrontal cortex
PPC: Posterior parietal cortex
FPN: Frontal-parietal network
PCC: Posterior cingulate cortex
PCu: Precuneus
ACC: Anterior cingulate cortex
mPFC: Medial prefrontal cortex
DMN: Default mode network
AI: Anterior insula
SN: Salience network
ECN: Executive control network
VLPO: Ventrolateral preoptic region
TMS: Transcranial magnetic stimulation
MNS: Median nerve stimulation
VNS: Vagus nerve stimulation
taVNS: Transcutaneous VNS
DBS: Deep brain stimulation
SCS: Spinal cord stimulation
